# Examining older adults’ vulnerability to online health scams: insights from routine activity theory

**DOI:** 10.3389/fpubh.2025.1585851

**Published:** 2025-04-30

**Authors:** Hongliang Chen, Miao He, Xiaowen Xu, David Atkin

**Affiliations:** ^1^College of Media and International Culture, Zhejiang University, Hangzhou, China; ^2^College of Communication, Butler University, Indianapolis, IN, United States; ^3^Department of Communication, University of Connecticut, Storrs, CT, United States

**Keywords:** routine activity theory, online health fraud, exposure to fraud, fraud victimization, older adults

## Abstract

**Introduction:**

Online health fraud has emerged as a significant digital risk for older adults in China, leading to considerable financial losses. This study aims to investigate the mechanisms underlying health fraud targeting and victimization among older adult populations, refining Routine Activity Theory (RAT) to account for the distinct stages of fraud exposure and victimization.

**Methods:**

A survey was conducted among retired individuals in July 2022. After removing incomplete responses, the final sample consisted of 471 participants. The survey assessed digital behavior patterns, exposure to promotional messages, health conditions, and fraud-related experiences.

**Results:**

The findings revealed that older adults who installed numerous mobile applications, frequently used social networking sites, and engaged in risky online behaviors were more likely to be targeted by health fraud. Additionally, exposure to promotional messages—both online and offline—and the presence of chronic diseases were significantly associated with increased fraud exposure. Regarding victimization, younger family intervention was found to mitigate financial losses, whereas older adults with limited digital protection skills experienced a higher likelihood of falling victim after exposure.

**Discussion:**

This study refines the Routine Activity Theory by conceptualizing online health fraud as a two-stage process: exposure and victimization. The results highlight both digital behavior and offline contextual factors in shaping fraud vulnerability among older adults. The findings contribute to the theoretical understanding of cybercrime targeting the older adult and offer practical insights for designing preventive interventions tailored to this vulnerable population.

## Introduction

The Internet enables older adults to stay connected with loved ones, purchase goods at lower prices, and access information easily and quickly (1). On the other hand, the Internet poses significant risks to older users, where fraudsters can obtain and exploit personally identified information about old online users, such as names, phone numbers, locations, or social media accounts, for deceptive purposes (2, 3). Online health fraud is among the most common scams targeting seniors. These fraudsters, often disguised as authorized doctors or legitimate drug retailers, sell ordinary foods with no medical benefits as healthcare products or prescriptions to naive victims (4). In the early stage of the COVID-19 pandemic, criminals exploited the older adults’ fears surrounding the virus to sell bogus drugs to them (61). Falling prey to health fraud can result in monetary loss and delay proper treatments for health risks.

Older adults are highly vulnerable to health scams because they place a high value on their physical health and have a strong desire to slow down the aging process. Older people are less tech-savvy and have lower literacy levels to detect false medical claims than younger individuals (5, 6). It can be challenging for them to identify fraudulent activities in the anonymous online environment. Meanwhile, many Internet platforms lack age-appropriate functions, further complicating the online experience for older people (7). Moreover, most older people live separately from their children, leaving them without their family’s help or protection (8). Although older people are at a greater risk of falling victim to online health scams, there is a lack of research examining the behavioral, technical, and social factors that contribute to their vulnerability.

This study aimed to develop a model that elucidates the roles of older adults’ online activities, habits, and personal and social factors in predicting their exposure to and victimization from online health fraud. We extended the original routine activity theory (RAT) by introducing a two-stage model: initial fraud exposure followed by victimization losses. Given the pervasive nature of risk exposure on the Internet, we investigated the moderating roles of young family members’ mediation and older adults’ technical protections in mitigating such risk. This extended model provides a more refined understanding of RAT in the online environment, particularly for vulnerable populations, and sheds light on key factors that warrant the attention of policymakers and health practitioners that can inform the design of campaigns and educational programs to combat such fraud.

## Literature review

### Routine activity theory

Routine activity theory (RAT) was proposed to explain the causes and patterns of crime in everyday life (9). According to RAT, three factors must come together in both time and space for direct-contact predatory crime to occur: a motivated offender, a suitable target, and the absence of a capable guardian (10). A suitable target refers to any individual or property that is valuable, visible and accessible. At the same time, a capable guardian can deter crime via legal authorities, surveillance cameras, or neighborhood watch groups (11). RAT has proven useful in analyzing street crimes and helps develop effective crime prevention strategies (12).

When applying RAT to the online context, it is essential to recognize that offenders can target potential victims at any time and place (13). Our study proposes that online health fraud can be interpreted as a two-stage process: exposure to fraud and actual victimization. The first stage, exposure to online health fraud, indicates whether fraudsters target an individual. An older adult who receives phishing emails, fraudulent phone calls, or promotions for a healthcare product is a likely target for scammers. As personal information is ubiquitous on social media, e-commerce platforms, and search engines, fraudsters can use digital data to identify older adult targets with higher pension income and poorer health status (14). The Internet allows fraudsters to repeatedly send phishing emails or messages at low cost (15).

The second stage involves determining whether an older user has lost money to online health fraud after exposure (16). According to RAT, effective guardianship is vital to deter potential crime. Unfortunately, current government regulations and Internet platform measures are insufficient to protect people from online scams (17). The rapid evolution of tactics and technologies used by fraudsters, inadequate legal frameworks and enforcement mechanisms to address online fraud, and limited resources to address scam issues pose challenges for government agencies working to prosecute online scams. Moreover, protective functions available from Internet providers are often too complicated for older users to use and cannot protect user privacy as expected (18). Since exposure to online scams is inevitable, it is critical to enhance older individuals’ self-protection skills and provide support from younger family members. Our study aims to utilize the RAT framework to investigate the relationship between older individuals’ online behaviors and their vulnerability to online fraud, as well as the efficacy of certain guardians in preventing online fraud victimization.

### Online routines and health fraud targeting

According to RAT, regular online routines create a digital convergence space where older users may inadvertently encounter potential offenders. Research on RAT has suggested that older adults’ increased engagement in various online activities, such as chatting, searching, entertainment, and payment, heightens their susceptibility to motivated offenders (19). Additionally, some Internet applications feature weaker security measures that can lead to data breaches for fraudulent purposes. As apps use algorithms to personalize content based on user’s browsing history, scammers can take advantage of private data to tailor their scams to satisfy the specific interests of potential targets (20). For instance, a fraudster could use stolen data from older users’ health-tracking apps to offer a miracle cure for a particular health condition (21). Moreover, older users tend to be trusting and assume that the information received online is legitimate. As an increasing number of older users become reliant on Internet services (6), they are more likely to click on links or download attachments without thinking through potential risks, which increases their exposure to motivated offenders. In this study, we assume that the more apps that an older person uses, the greater the likelihood that fraudsters can obtain personal information and exploit it to target them with health fraud. More formally:

*H1: Among the old users, the number of applications used is positively related to risk of exposure to online health fraud.*

Social network services (SNSs) have attracted many older users. As many young individuals migrate to big cities for better job opportunities, social media provides a convenient way for older users to stay connected with their children (22). In addition to intra-family communication, social media enables older individuals to find others who share similar interests and hobbies (23). Joining groups with common interests can provide a sense of community and belonging for old users who may feel isolated. Entertainment-seeking is another crucial driver of SNS use among older people. Social media in China offers various entertainment options, including videos, games, and live streaming that older people can use to pass the time. Many older users use social media to access information about health and wellness, including tips for staying healthy and managing chronic conditions (24).

Despite these benefits, routine uses of SNSs also raise significant privacy concerns. To access these platforms, users must agree to a range of privacy provisions. Unfortunately, those unfamiliar with privacy protection may inadvertently disclose sensitive personal information or leave the privacy settings at “default” (25). Cybercriminals can easily collect private data targeting seniors via a specific profile with health problems and higher pensions (26). The regulation of social media platforms thus remains inadequate, allowing offenders to hide their true identities behind fake profiles to deceive victims (27). Therefore, we propose that increased social media use should lead to greater health fraud exposure among old adults; that is:

*H2: Among older users, SNS use is positively related to exposure to online health fraud.*

Older victims are often tricked into transferring money to fake businesses or buying counterfeit products in a health fraud event. Online payment systems allow for quick and easy transactions, but in the meantime, these systems require users to share personal and financial information, which increases the risk of online fraud (28, 29). Fraudsters utilize the anonymous and instant features of online payment to make money and quickly evade detection. The transactions on fake websites can lead to stolen financial accounts and monetary losses (30). Compared to traditional payment methods, it is more difficult to verify the identity of the money receiver. In online fraud, many older victims transfer money to unfamiliar accounts, prompting significant losses.

Currently, online payment platforms still lack sufficient security measures to protect users from cyberattacks. For example, some online payment providers have not implemented two-factor authentication nor strong password requirements, making it easier for fraudsters to steal user accounts. Older users who use weak passwords and auto-save the passwords online are more susceptible to account breaches. Additionally, older victims are less likely to report fraudulent activities due to the embarrassment that results in unreported online fraud cases. We thus propose that older adults’ online payments increase their likelihood of being targeted by health scam content. More formally:

*H3: Among the older users, online payment use is positively related to exposure to online health fraud.*

Online shopping represents another popular online routine for older individuals in China to purchase a wide range of products, including medical and healthcare items (31). As individuals age, they are likely to seek out health-related products online. But many of them experience difficulty in identifying scams, especially those that appear legitimate (32). Previous studies found that online shopping carries the risk of privacy theft, which increases the likelihood of experiencing fraud loss (33, 62).

In particular, cybercriminals can exploit this risk by creating counterfeit retail webpages, advertising or selling fake healthcare products, and stealing financial information during transactions. Compared to other merchandise, healthcare products are generally more expensive and require professional knowledge that many seniors need. Thus, we assume that online shopping may be a high-risk factor prompting seniors to be trapped in fraud. More formally:

*H4: Among the older users, online shopping is positively related to exposure to online health fraud.*

### Risky encounters and health fraud targeting

Engaging in risky Internet behaviors, such as clicking on unfamiliar links and downloading free software from unknown sources, can result in the accidental installation of malicious programs (63). These programs garner personal information, such as login credentials, physical addresses, and credit card numbers, that criminals can use for scam purposes. Limited computer skills, combined with age-related physiological and psychological changes, make older adults less sensitive to risky Internet use (34). Research has shown that older users are more vulnerable to accidentally clicking on fraudulent websites and downloading malicious software (35). Such risky Internet uses can facilitate unauthorized access to user data and enable fraudsters to create personalized phishing information to a target’s interests (36). Drawing from this assumption, we posit that:

*H5: Among older users, risky Internet use is positively related to the risk of exposure to online health fraud.*

In addition to online routines, exposure to healthcare promotional ads—whether online or offline—can increase the risk of healthcare fraud targeting older people. Fraudulent healthcare products are often advertised using exaggerated claims, false promises, and manipulated testimonials, which are extremely difficult to identify (37, 38). Fraudsters may even create fake licenses and hire phony experts to build credibility. Compared to online sources, seniors may perceive promotional messages from television or phone calls as more professional and trustworthy. Offline promotional ads often provide more detailed product descriptions than their online counterparts and may direct customers to complete transactions online. Fraudsters can collect the personal data of seniors through marketing calls, allowing them to evaluate the targets’ profiles and improve the design of phishing messages (39). Based on the theory and research reviewed above, then, we propose that:

*H6: Among the older users, exposure to promotional messages is positively related to exposure to online health fraud.*

### Suitable targets

In the context of online health fraud, seniors in poorer health are suitable targets, as they often face chronic health conditions that cause discomfort, so they strongly desire better health and longevity (40, 41). They are likely to fall into fraudulent schemes that masquerade spokespeople as medical professionals and promise effective treatment for their ailments (37). Due to mobility limitations and poor health conditions, older adults are more inclined to seek health information online. However, they may lack the ability to distinguish between genuine and fraudulent information, which makes them more vulnerable to falling into the health deception traps set by fraudsters. We propose that, burdened by chronic diseases, older adults become more desperate to find solutions that render them ideal targets of health fraud. More formally:

*H7: Among the older users, chronic disease burden is positively related to exposure to online health fraud.*

### Health fraud victimization

The Internet has created a favorable environment for fraudsters to defraud older adults after exposure. In particular, online health fraud involves deceiving seniors into transferring money to fraudsters without consulting family members or financial institutions. The consequences of online health fraud victimization can be disastrous for older people, resulting in significant economic losses, severe physical and psychological harm, and even conflicts in family relationships (42, 43). Unfortunately, due to cognitive decline, most older individuals experience difficulty discerning between legitimate and fraudulent messages. Moreover, due to feelings of isolation and loneliness, the assistance and companionship offered by fraudsters can be alluring to older adults, ultimately increasing their likelihood of falling victim to fraud. We thus propose that:

*H8: Among older users, exposure to online health fraud is positively related to the risk of health fraud victimization.*

### Potential moderators influencing health fraud outcomes

As online fraud exposure remains a pervasive concern on the Internet, it is critical to understand how to protect older netizens from victimization. Prior research established that two guardianship forms—social and physical guardianship—effectively deter crime events (44). According to RAT, social guardians include friends, neighbors, and family members (45), while technological protections like security software and complex account passwords are useful physical guardians. Family members are impactful social guardians in protecting older people from health fraud victimization. Young family members can evaluate the legitimacy of suspicious offers and make informed health decisions for their old family members (46); they can also monitor online activities and block transactions with unknown retailers.

Extant studies have identified two primary strategies to mitigate negative technology influence: active mediation and restrictive mediation (47). Active mediation involves young family members discussing media content with old family members and guiding them on how to use the Internet appropriately (48). When exposed to fraudulent information, these conversations can make older users pay attention to risky information, increasing their alertness to protect their privacy, accounts, and properties. Restrictive mediation involves regulating older adults’s Internet use by setting rules and restrictions to monitor their media use (49). Young family members can track money transfers, restrict unsafe app downloading, and avoid online interactions with or purchases from suspicious sources. Based on the literature and theory reviewed above, we propose that active and restrictive interventions for young family members can reduce the likelihood of victimization of older adults to fraudulent messages. In particular:

*H9: Among younger family members, active mediation negatively moderates the effect of older adults’ exposure to online health fraud on online health fraud victimization.*

*H10: Among younger family members, restrictive mediation negatively moderates the effect of older adults’ exposure to online health fraud on online health fraud victimization among older users.*

Aside from social guardianship, physical guardianship also plays an important role in preventing health fraud victimization among older individuals. For example, installing antivirus software significantly reduces the risk of malware infection. Extant research found that sophisticated password combinations and privacy settings can prevent older adults from falling prey to privacy breaches (50). However, most older users lack sufficient digital skills or resources to implement protective measures. This leaves such online behaviors unsecured. Therefore, the adverse impact of fraud targeting on the old adults’ vulnerability to victimization may be greatly aggravated. More formally:

*H11: Among the older users, lack of technical protection positively moderates the effect of exposure to online health fraud on online health fraud victimization.*

**Figure 1:**
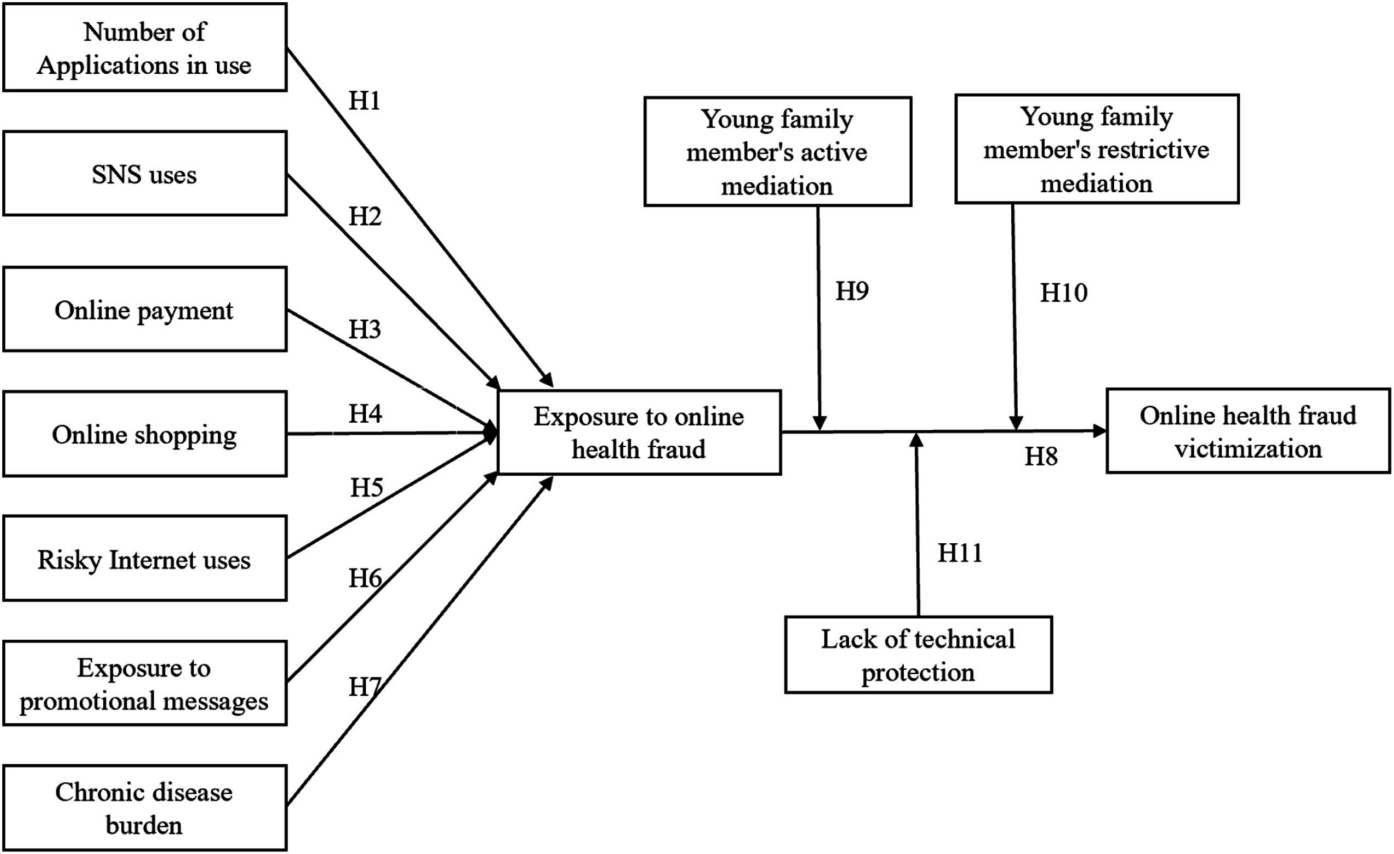
Proposed model of the current study.

The proposed framework of this study is presented in Figure 1.

## Methods

### Sampling procedure

We collected survey data in July 2022, targeting older retirees. The study design was approved by the P.I.’s Institutional Review Board (anonymized for review purposes). Given that retirement marks a significant transition for most older Chinese adults, bringing changes such as a reduced social circle, increased spare time, and decreased income (e.g., the loss of “accumulation fund”), their Internet engagement tends to increase. Therefore, we included participants above the retirement age threshold—55 for females and 60 for males. To ensure the accuracy of the survey results, we recruited over 500 students and faculty volunteers from a university in southeast China. We asked them to find family members in the qualified age range. After deleting the incomplete results, the final valid sample was 471.

Table 1 displays the descriptive statistics for our sample. The average age of participants was 62.06 years, and most lived separately from their children (66.45%). Participants reported an average annual income between 50,001 and 100,000 RMB. Regarding educational attainment, 41.83% of participants had earned a bachelor’s degree or above.

**Table 1:** Descriptive statistics of demographic variables (*N* = 471).

Variables	Mean/percentage	SD	Min	Max
Gender (male)	33.12%			
Age	62.06	5.66	55	86
Education	4.10	1.12	1	7
1 = no education experience	1.06%			
2 = elementary school	6.37%			
3 = middle school	30.57%			
4 = associate degree	20.17%			
5 = bachelor’s degree	36.73%			
6 = graduate’s degree	4.46%			
7 = doctor’s degree	0.64%			
Household income	3.76	1.92	1	8
1 = below 20,000 RMB	9.34%			
2 = 20,001–50,000 RMB	16.35%			
3 = 50,001–100,000 RMB	29.72%			
4 = 100,001–150,000 RMB	15.92%			
5 = 150,001–200,000 RMB	8.92%			
6 = 200,001–250,000 RMB	8.49%			
7 = 250,001–300,000 RMB	4.03%			
8 = Above 300,001 RMB	7.22%			
Living status (living with children)	33.55%			

### Measurement

#### Exposure to online health fraud and online health fraud victimization

Adapted from existing studies (41), we used six items to measure exposure to online health fraud. The participants were asked whether they had been exposed to the following product information online: (1) unlicensed healthcare products; (2) medical devices with false claims; (3) miracle drugs for curing chronic diseases; (4) beauty and weight loss products that failed to deliver promised results; (5) exaggerated medical measures; and (6) unscientific folk remedies. Participants rated their frequency of exposure on a scale ranging from 1 = never to 5 = always (*M* = 2.25, *SD* = 1.13; Cronbach’s *α* = 0.93). Using the same items, we then asked how much money they lost due to relevant health fraud. Response categories for online health fraud victimization included: 0 = “no monetary loss,” 1 = “less than 500 RMB,” 2 = “500–999 RMB,” 3 = “1,000–4,999 RMB,” 4 = “5,000–9,999 RMB,” 5 = “more than 10,000 RMB” (*M* = 0.59, *SD* = 1.06). Some 31.21% of participants had experienced monetary losses due to online health fraud, with 0.85% reporting losses exceeding 10,000 RMB.

#### Number of applications in use

We assessed the number of web applications on participants’ devices by asking respondents to report the number of apps utilized. The answer was measured with five categories: 1 = less than 10, 2 = 11–20, 3 = 21–40, 4 = 41–70, and 5 = more than 71. Most participants (95.54%) have fewer than 40 apps on their mobile phones (*M* = 1.97, *SD* = 0.89).

#### SNS uses

In line with previous research (51), we measured SNS uses by asking respondents to estimate the frequency of their use of platforms, such as TikTok and Weibo, on a scale ranging from 1 = never to 5 = always (*M* = 3.45, *SD* = 1.41).

#### Online payment

Adapted from existing research (51), we used a single item to measure one’s engagement with online payment. The participants were asked to indicate how often they used mobile payment apps like Alipay and WeChat to complete online transactions on a scale, ranging from 1 = never to 5 = always (*M* = 4.03, *SD* = 1.32).

#### Online shopping

Following previous research (36), we assessed five types of online shopping behaviors: e-commerce platform shopping, live-streaming platform shopping, online membership purchase of video, music, e-reading and newsfeed services, group-buying platform shopping, and online peer-to-peer shopping. Respondents rated their frequency of involvement in each behavior on a scale ranging from 1 = never to 5 = always. The individual responses for each type of online shopping behavior were averaged to create an overall score for online shopping (M = 2.43, *SD* = 0.93, Cronbach’s *α* = 0.80).

#### Risky internet uses

Following previous research (52), the current study measured risky Internet uses through a five-item scale. Respondents were asked to indicate their engagement in risky behaviors like “click on unfamiliar links,” “click on pop-ups,” “download free software,” “download free music, movies, and videos,” and “download unknown files.” Responses to each measure ranged from 1 = never to 5 = always (*M* = 2.03, *SD* = 0.77; Cronbach’s *α* = 0.78).

#### Exposure to promotional messages

We utilized four items to measure exposure to promotional ads. Respondents reported how often they watched television shopping programs, browsed ads online, viewed ads pushed by apps, and responded to marketing phone calls in daily life. Responses were given from 1 = never to 5 = always (*M* = 2.45, *SD* = 0.99, Cronbach’s *α* = 0.82).

#### Chronic disease burden

To measure the respondents’ chronic disease burdens, 11 chronic diseases were listed, including heart disease, stroke, cancer, arthritis, osteoporosis, chronic obstructive pneumonia, diabetes mellitus, chronic nephritis, chronic kidney failure, digestive system diseases, and oral diseases. The participants were asked whether they suffered from each disease, with responses coded dichotomously. The sum of responses was used to create an index of chronic disease burden (*M* = 1.03, *SD =* 1.06).

#### Young family members’ active mediation

Adapted from previous research (47), we used four items to measure young family members’ active mediation. The items included “my children or other relatives told me not to disclose private information,” “…interpreted the dangers of using the Internet to me,” “…reminded me not to answer unfamiliar calls,” and “…discussed anti-fraud strategies.” Responses were given using a 4-point Likert scale, ranging from 1 = totally disagree to 4 = totally agree (*M* = 3.30, *SD* = 0.62; Cronbach’s *α* = 0.80).

#### Young family members’ restrictive mediation

Emulating prior research (47), we used four items to measure young family members’ restrictive mediation, including “children managed my mobile payment accounts,” “children made rules about using the Internet for me,” “children limited online shopping behaviors of mine,” and “children limited my behaviors when using the Internet.” The answer was given on a 4-point Likert scale, ranging from 1 = totally disagree to 5 = totally agree (*M* = 2.19, *SD* = 0.91, Cronbach’s *α* = 0.91).

#### Lack of technical protection

We adopted four items to measure respondents’ inability to use protective technologies (53). The items are: (1) “I installed and regularly updated security software on my devices,” (2) “I set passwords for my devices and accounts,” (3) “I regularly check the viruses on my devices,” and (4) “I made privacy settings to restrict the websites from misusing my data.” Responses ranged from 1 = totally disagree to 5 = totally agree. We reverse-coded this variable so that higher scores indicate weaker protective behaviors (*M* = 2.07, *SD* = 0.73; Cronbach’s *α* = 0.79) (see Table 2).

**Table 2:** Descriptive statistics of independent and dependent variables (*N* = 471).

Variables	Mean/percentage	SD	Min	Max	Cronbach’s *α*
Exposure to online health fraud	2.25	1.13	1	5	0.93
Online health fraud victimization	0.59	1.06	0	5	
0 = No monetary loss	68.79%				
1 = less than 500 RMB	15.29%				
2 = 500–999 RMB	7.22%				
3 = 1,000–4,999 RMB	6.16%				
4 = 5,000–9,999 RMB	1.70%				
5 = More than 10,000 RMB	0.85%				
Number of applications in use	1.97	0.89	1	5	
1 = less than 10	36.60%				
2 = 11–20	34.47%				
3 = 21–40	24.47%				
4 = 41–70	4.26%				
5 = more than 71	0.21%				
SNS uses	3.45	1.41	1	5	
1 = never	11.68%				
2 = seldom	19.32%				
3 = sometimes	13.16%				
4 = often	23.78%				
5 = always	32.06%				
Online payment	4.03	1.32	1	5	
1 = never	6.79%				
2 = seldom	12.31%				
3 = sometimes	7.64%				
4 = often	17.41%				
5 = always	55.84%				
Online shopping	2.43	0.93	1	5	0.80
Risky internet uses	2.03	0.77	1	5	0.78
Exposure to promotional messages	2.45	0.99	1	5	0.82
Chronic disease burden	1.03	1.06	0	11	
Young family members’ active mediation	3.30	0.62	1	4	0.80
Young family members’ restrictive mediation	2.19	0.91	1	4	0.91
Lack of technical protection	2.07	0.73	1	5	0.79

### Analytical strategy

We utilized structural equation modeling (SEM) and ordinary least-squared regressions to test the proposed model with Stata 15. The SEM fitness was assessed using the following threshold values: Root Mean Square Error of Approximation (RMSEA) < 0.06, Comparative Fit Index (CFI) > 0.95, Standardized Root Mean Squared Residual (SRMR) < 0.05 (54). Exogenous variables--such as gender, age, educational level, household income, and living status--were added as controls. The model demonstrated an excellent fit with the data: χ ^2^(25) = 415.49, *p* < 0.001; RMSEA = 0.056; CFI = 0.978; SRMR = 0.010. The moderating effects of guardianship from family and themselves were assessed by a series of moderation models, holding constant for gender, age, educational level, household income, and living status.

## Results

**Figure 2:**
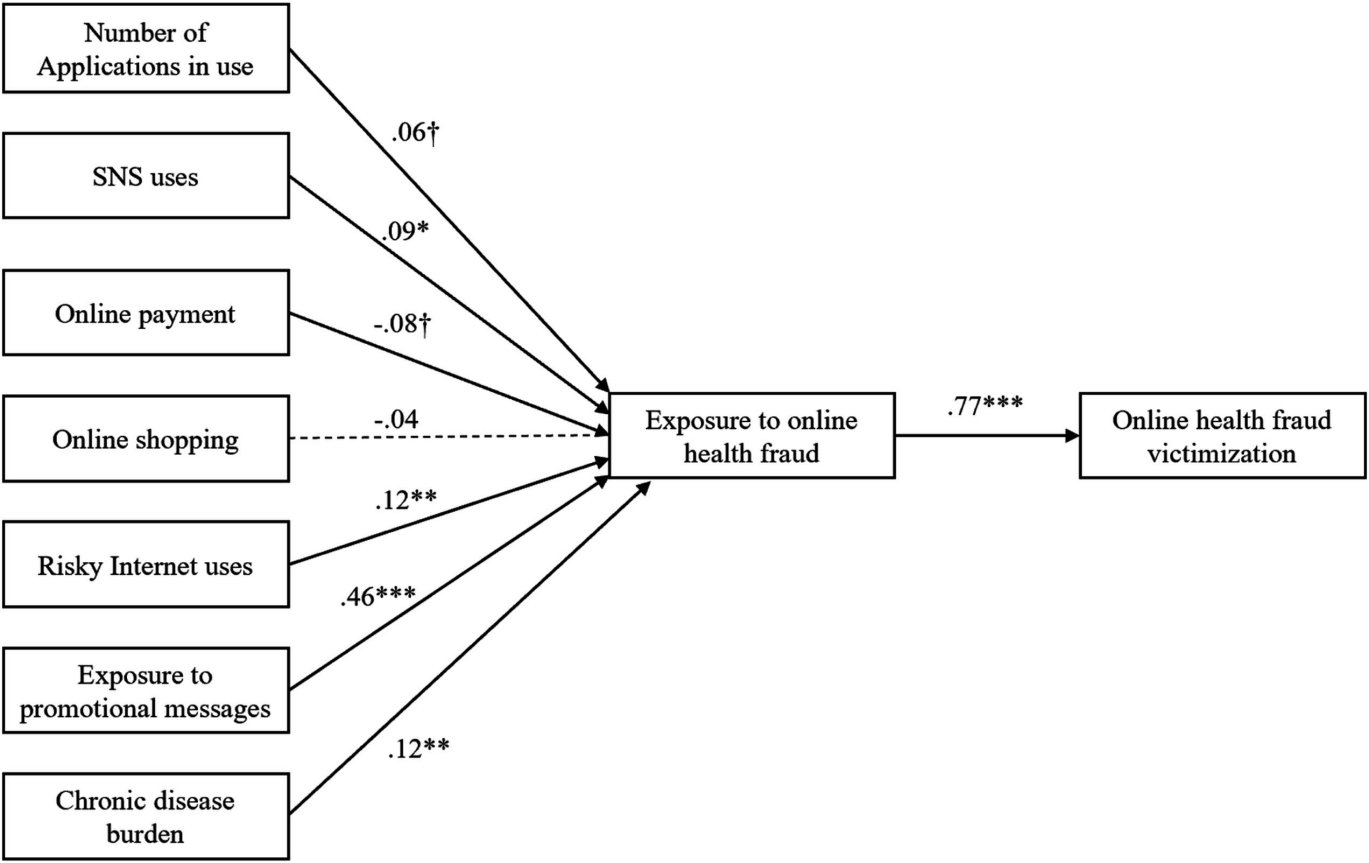
Results of structural equation model. ^†^*p* < 0.10, *p < 0.05, **p < 0.01, ***p < 0.001.

Hypotheses 1–8 were tested using structural equation modeling, and the results are presented in Table 3 and Figure 2. H1 posits that the number of applications in use by older adults is associated with a higher risk of exposure to online health fraud. The results indicated that the effect was positive and significant (*β* = 0.06, *p* < 0.10), providing marginal support for H1. According to H2, SNS use was positively associated with online health fraud exposure. The findings uncovered a significant positive effect (*β* = 0.09, *p* < 0.05). Hence, H2 was supported.

**Table 3:** Results of path coefficients pertinent to H1–H7.

Hypotheses	DV = Exposure to online health fraud	*β*	Results
H1	Number of applications in use	0.06^†^	Marginally supported
H2	SNS uses	0.09*	Supported
H3	Online payment	−0.08^†^	Rejected
H4	Online shopping	−0.04	Rejected
H5	Risky Internet uses	0.12**	Supported
H6	Exposure to promotional messages	0.46***	Supported
H7	Chronic disease burden	0.12**	Supported
	Gender	0.09*	
	Age	−0.08^†^	
	Education	−0.07^†^	
	Income	0.01	
	Living status	−0.08*	

^†^*p* < 0.10, *p < 0.05, **p < 0.01, ***p < 0.001.

H3 postulates that older individuals’ level of engagement with online payment increases their exposure to online health fraud. As shown in Figure 2 and Table 3, the effect of online payment was marginally significant (*β* = −0.08, *p* < 0.10), but the direction is contrary to H3. Hence, H3 was rejected. H4 posited that involvement in online shopping is related to online health fraud exposure. Per Table 3, the effect of online shopping on fraud exposure was insignificant (*β* = −0.04, *p* = 0.45), leaving H4 without support.

H5 holds that risky Internet uses increase the likelihood of online health fraud exposure. The effect was positive and significant (*β* = 0.12, *p* < 0.01), supporting H5. H6 posited that older adults’ exposure to promotional messages is related to a higher risk of exposure to online health fraud. Study results support this prediction, showing a significant positive effect (*β* = 0.46, *p* < 0.001), supporting H6.

H7 suggested that those with heavier chronic disease burdens are more likely to be targeted by fraudsters. The results indicated that the effect of chronic disease burden was positive and significant (*β* = 0.12, *p* < 0.01), supporting H7. H8 assumed that exposure to online health fraud is positively related to online health fraud victimization. The results in Table 4 revealed that the effect was positive and significant (*β* = 0.77, *p* < 0.001), which supported H8.

**Table 4:** Results of path coefficients pertinent to H8.

Hypothesis	DV=Online health fraud victimization	*Β*	Results
H8	Exposure to online health fraud	0.77***	Supported
	Gender	0.01	
	Age	0.01	
	Education	−0.00	
	Income	0.08^†^	
	Living status	0.06	

^†^*p* < 0.10, *p < 0.05, **p < 0.01, ***p < 0.001.

To examine the mediating role of exposure to health fraud in the relationship between the independent variables and victimization loss, we conducted a series of mediation analyses. As shown in Table 5, SNS use, risky Internet behaviors, exposure to promotional messages, and chronic disease burden were all found to increase victimization loss through the mediating effect of fraud exposure.

**Table 5:** Results of mediation tests.

Mediation paths	Estimates
Number of Applications in use→Exposure to online health fraud→Online health fraud victimization	0.05^†^
SNS uses→Exposure to online health fraud→Online health fraud victimization	0.04*
Online payment→Exposure to online health fraud→Online health fraud victimization	−0.02
Online shopping→Exposure to online health fraud→Online health fraud victimization	−0.04
Risky Internet uses→Exposure to online health fraud→Online health fraud victimization	0.08*
Exposure to promotional messages→Exposure to online health fraud→Online health fraud victimization	0.29***
Chronic disease burden→Exposure to online health fraud→Online health fraud victimization	0.06**

^†^*p* < 0.10, *p < 0.05, **p < 0.01, ***p < 0.001.

Next, we tested the moderating roles of guardianship acts. The results related to H9–H11 can be found in Table 6. Results of H9 and H10 indicated that young family members’ active mediation negatively moderated the effect of fraud exposure on fraud victimization (*β* = −0.80, *p* < 0.01). In contrast, the effect of young family members’ restrictive mediation is not significant (*β* = −0.13, *p* = 0.38). Regarding H11, moderating effect of lack of technical protection was found significant (*β* = 0.46, *p* < 0.001), indicating that lack of technical protection positively moderated the effect of exposure to online health fraud on victimization. Hence, H11 was supported.

**Table 6:** Standardized regression coefficients of the moderation effect pertinent to H9–H11.

DV=Online health fraud victimization		Model 1	Model 2	Model 3	Hypotheses
Exposure to online health fraud (A)		1.19***	0.54***	1.19***	
Young family members’ active mediation (B)		0.17*			
Young family members’ restrictive mediation (C)			0.09		
Lack of technical protection (D)				−0.16^†^	
A*B (H9)		−0.80**			Supported
A*C (H10)			−0.13		Rejected
A*D (H11)				0.46***	Supported
Sex		0.07	0.08^†^	0.08^†^	
Age		−0.06	−0.05	−0.06	
Education		−0.06	−0.05	−0.05	
Income		0.09^†^	0.10*	0.10*	
Living status		0.05	0.03	0.04	
Adj R^2^		0.25***	0.23***	0.26***	

^†^*p* < 0.10, *p < 0.05, **p < 0.01, ***p < 0.001.

Focusing on individual characteristics (Tables 3, 4), findings suggest that males (*β* = 0.09, *p* < 0.05) and those living apart from children (*β* = −0.08, *p* < 0.05) were more likely to be targeted by fraudsters. The inverse relationship between educational level and fraud exposure was significant (*β* = −0.07, *p* < 0.10), and although the effect was modest, individuals with lower education faced an increased likelihood of online fraud exposure. After being targeted, older adults with higher income were likely to lose more money due to online health fraud (*β* = 0.09, *p* < 0.05). These findings were consistent with prior research (55, 56).

## Discussion

This study is among the first to investigate predictors of exposure to and victimization of online health fraud among older adults in China. We extended routine activity theory (RAT) by examining the two stages of online health fraud: exposure to fraud and victimization. The current framework enhanced RAT by providing insights into the technical, social, and behavioral mechanisms influencing older adults’ susceptibility to online health fraud. Additionally, our research uncovered the critical moderating roles of family members’ mediation and lack of technical protection in victimization. Although exposure to scams may be unavoidable, these factors significantly impact the financial losses incurred, enriching RAT by incorporating individual vulnerabilities into the process of healthcare fraud victimization.

In particular, our research findings indicated that older adults’ engagement in routine Internet activities and risky encounters increased their exposure to health fraud content, further enriching the RAT framework. Moreover, older individuals with severe health conditions are more likely to be targeted, highlighting the need to consider victims’ personal characteristics in cybercrime studies. Moreover, we found that after exposure, capable guardianships from family members and older adults could deter fraud crime and reduce financial losses. These results thus shed light on the importance of personal habits and family intervention in preventing health fraud exposure and victimization. By exploring the two stages of online fraud, our study provides a comprehensive understanding of the conditions for online health fraud against older adults and inspires contemplation on how it can be prevented.

Moreover, current findings indicate that SNS usage is the riskiest among different online routines for the older adults. One possible explanation is that SNS apps require users to share personal information with online contacts, exposing them to a heightened risk of fraud targeting. Older users lack the necessary knowledge and skills and cannot effectively identify and respond to online fraud attempts. SNS platforms should thus enhance their data privacy policies to protect the security of their users, particularly older individuals. Essential remedies include better controls for users’ privacy settings, more robust security measures, and clear descriptions of user data usage.

Contrary to extant research (19, 57), online payment and shopping did not significantly increase health fraud exposure among old users. This finding suggests that payment and e-commerce platforms may have implemented adequate measures to address the threat of online fraud. For example, many mobile payment platforms have built-in features that allow users to monitor suspicious messages and transactions and quickly block unauthorized payments. E-commerce platforms provide consumers with tools to protect their rights, such as customer service support and dispute resolution services (58). Other online service providers should learn from the defensive mechanism implemented by online payment and shopping platforms to reduce the risk of online health fraud against their older customers.

According to RAT, the convergence of motivated offenders and vulnerable targets is essential for crimes to occur. This study highlighted that digital convergence emerged through various risky Internet uses, such as clicking on suspicious links or downloading unverified software, which significantly increased the likelihood of fraud exposure. Additionally, findings indicate that exposure to online and offline promotional ads puts old individuals in danger of health fraud targeting. Older individuals may be more willing to try new healthcare products to improve their health due to their deteriorating health. Compared to younger individuals, older users tend to be trusting. They may not question health retailors’ authenticity, which gives fraudsters chances to collect private information via promotional calls and pop-up ads (43). Past work revealed that fraudsters often display a caring attitude and attempt to create an intimate relationship with older victims, which can be alluring to those who live alone (59). To reduce health fraud targeting, providing older users with appropriate guidance on the potential harms of risky online encounters is crucial.

Consistent with RAT, this study found that older adults with multiple chronic diseases are vulnerable targets for online health fraud. As frequent purchasers of health products online, older consumers are likely to leave many personal records online that fraudsters can manipulate. To address this issue, media outlets, community organizations, and family members should collaborate to provide seniors with effective health consultations and help them detect the risks of purchasing health products online. Furthermore, this study documented that respondents who were male, living apart from their children, and had higher incomes were more likely to be targeted by health fraudsters. This underscores the crucial role of family support, as young family members can assist older adults in filtering and verifying information, providing decision-making and emotional support, and offering technical guidance, thereby reducing the risk of fraud victimization. For older adults with chronic illnesses, social support becomes even more critical, as they are more focused on their health conditions and rely heavily on the Internet for health-related information. In such cases, family members can help enhance their access to legitimate medical resources.

Next, our study uncovered a positive relationship between exposure to online health fraud and fraud victimization. Consistent with RAT, this study confirmed that effective guardianship represents a pivotal deterrent to health fraud victimization. In particular, we found that active mediation from young family members is more effective in reducing older people’s loss of victimization, compared to restrictive mediation. As Chinese cultural norms value older parents as authorities in the family (60), restrictive mediation may trigger resistance among older adults, leading them to perceive a deprivation of autonomy. This could drive them to conceal their online activities from family members, increasing their susceptibility to the “warm traps” set by fraudsters. To prevent health-related scams, it is imperative for adult children to adopt appropriate communication strategies—including regular discussions about health information, emotional support, and guidance through suggestions—rather than directives during interactions.

Our study also demonstrated that older users’ lack of technical protection was positively related to a higher victimization loss after being targeted. As fraudsters continually refine their tactics, technical protection measures such as antivirus software and firewalls are essential in reducing the fraud risk. However, the majority of older users need to gain such skills. Current findings also suggested that a multi-faceted approach is necessary to address the complex issue of older adult health fraud exposure. By enhancing privacy policies, providing education and support, and promoting family and community involvement, we can better protect older individuals from online health fraud.

### Limitations

There are several limitations in this study. First, the present study used a cross-sectional survey design, which limited the capacity to draw causal inferences. Later work should consider collecting longitudinal data to establish time order. Moreover, a qualitative method like in-depth interviews would be helpful to gather detailed data on the experiences and perspectives of older individuals. Second, considering that our sample was drawn from the (grand)parents of university students and faculty members, the respondents were likely to have relatively high educational backgrounds. Although this sample could represent the older population with the highest levels of Internet engagement, it might overlook truly disadvantaged groups. Future research should focus on reaching a more diverse sample to enhance the generalizability of study findings. Third, this study measured fraud exposure and victimization through self-reported items, meaning that our results capture participations’ perceptions rather than concrete evidence of fraud victimization. Given that some respondents may overestimate or underestimate their financial losses for various reasons, future research should explore more objective indicators. Finally, as our study focused solely on older adults and did not include younger respondents in the sample, it limited the possibility of conducting comparative analyses across age groups. Future research would benefit from examining whether levels of fraud exposure and financial loss differ significantly between older and younger populations.

## Data Availability

The raw data supporting the conclusions of this article will be made available by the authors, without undue reservation.
